# Maternal Adverse Childhood Experiences and Child Socio‐Emotional and Behavioural Outcomes: A Systematic Review of Prenatal Mechanisms

**DOI:** 10.1111/ppe.70190

**Published:** 2026-08-29

**Authors:** Sara Avendano, Aicha Mourchidi, Susana Barbosa, Laetitia Davidovic, Barbara Heude, Cédric Galéra, Judith van der Waerden

**Affiliations:** ^1^ Social Epidemiology, Mental Health, and Addiction Team (ESSMA), Sorbonne Public Health Institute, INSERM Sorbonne University Paris France; ^2^ CNRS UMR7275, INSERM U1318, Institut de Pharmacologie Moléculaire et Cellulaire Université Côte d'Azur Valbonne France; ^3^ Obstetric, Perinatal, Paediatric Life Course Epidemiology (Oppale), Center for Research in Epidemiology and Statistics, INSERM, INRAE Paris Cité University and Sorbonne University Paris Nord Paris France; ^4^ Epidemiology, Development and Prevention of Mental Health Problems Using a Life Span Perspective (Healthy), Bordeaux Population Health Research Center, INSERM U1219 Bordeaux University Bordeaux France; ^5^ Charles Perrens Hospital Bordeaux France

**Keywords:** adverse childhood events, child development, life course, mental health, pregnancy

## Abstract

**Background:**

Pregnancy represents a crucial period for understanding key pathways contributing to the intergenerational effects of adverse childhood experiences (ACEs). However, prenatal mechanisms linking maternal ACEs to subsequent child mental health outcomes are still poorly understood compared to postnatal ones.

**Objective:**

To review the biopsychosocial mechanisms during pregnancy that may contribute to the relationship between maternal ACEs and child socio‐emotional and behavioural development.

**Data Sources:**

We systematically searched PubMed, PsycInfo and Web of Science from inception up to February 2026.

**Study Selection and Data Extraction:**

We included peer‐reviewed observational studies (cohort, case–control, cross‐sectional) evaluating prenatal biological, psychological, or social mechanisms linking maternal ACEs to child socio‐emotional/behavioural outcomes (Ages 0–18); qualitative studies and grey literature were excluded. Two reviewers independently screened studies and extracted data on study design, mediators and effect sizes, with disagreements resolved by a third reviewer.

**Synthesis:**

Narrative synthesis was used to summarise study characteristics, mediation pathways and analytical strategies in tabular form. Results were synthesised by type of prenatal mechanism.

**Results:**

Of 2641 records identified, 122 full texts were assessed and 13 studies met inclusion criteria. Prenatal maternal depression and anxiety emerged as the most frequently studied mechanisms, showing some evidence of independent effects irrespective of postnatal factors, but most of the findings point towards a ‘chain‐of‐risk’ model, where prenatal mental health interacts with the postnatal environment. Biomedical antepartum risks and maternal–fetal attachment also showed mediating effects, but without accounting for postnatal influences. The small number of eligible studies underscores the limited exploration of prenatal transmission pathways.

**Conclusions:**

Prenatal mental health difficulties, particularly in interaction with postnatal factors, may play a role in the intergenerational transmission of ACEs. Further studies using robust methods to disentangle prenatal and postnatal contributions are needed to inform targeted interventions.

## 1 Background

Research has consistently demonstrated the detrimental effects of adverse childhood experiences (ACEs) on adult physical and mental health [1, 2], as well as their potential effect on subsequent generations [3]. Maternal ACEs have been associated with offspring's socio‐emotional and behavioural difficulties [4, 5], outcomes that, if left unaddressed, could increase the risk for physical and mental health problems [6], and negatively impact child and family well‐being. Recent systematic reviews and meta‐analyses [5, 7, 8, 9] have summarised the evidence on the potential mechanisms linking maternal ACEs to these child outcomes. Key identified factors include postnatal maternal mental health [5, 7, 8], maternal insecure attachment [8], poor parenting practices [5, 8] and children's own exposure to maltreatment [7].

The Developmental Origins of Health and Disease (DOHaD) framework [10] suggests that negative exposures in utero can shape the later onset of chronic diseases, including mental health disorders [11]. Notably, several studies have reported associations between maternal childhood adversity and alterations in offspring brain development during fetal life [12] and in the early neonatal period [13, 14] prior to the influence of postnatal factors. Pregnancy may represent a sensitive period [15] during which maternal adversity effects could be transmitted to the next generation [16]. However, the contributing prenatal mechanisms remain poorly understood, underscoring the need to clarify how these pathways operate.

ACEs are associated with dysregulated physiological responses and psychopathology during pregnancy [14, 17, 18], which may directly disrupt fetal development. Indirectly, prenatal maternal mental health, along with the adaptation and preparation for motherhood, may influence the postnatal environment, including maternal postnatal mental health, mother–child interactions and potential maltreatment, all key determinants of children's socio‐emotional and behavioural development [19, 20]. Identifying the mechanisms that most strongly contribute to risk transmission is essential to breaking the cycle of adversity. In this context, mediation analyses appear to be strong methodological tools to explore the role of prenatal and postnatal factors and allow us to evaluate causal pathways between maternal ACEs and child mental health outcomes. Yet, to our knowledge, no systematic review has specifically examined the underlying mechanisms for the prenatal period, limiting our ability to develop a comprehensive understanding of intergenerational pathways.

The objective of this study is to review and summarise the existing literature on the prenatal biopsychosocial mechanisms that may contribute to the relationship between maternal ACEs and infant and child socio‐emotional and behavioural development.

## 2 Methods

We conducted a systematic search following the Preferred Reporting Items for Systematic Reviews and Meta‐Analyses (PRISMA) guidelines [21]. The corresponding protocol was registered in PROSPERO (CRD42023495297).

### 2.1 Study Search

Relevant peer‐reviewed articles were identified through comprehensive searches in PubMed, Web of Science and PsycINFO. In our search strategy, we used a combination of MeSH terms and keywords related to intergenerational or maternal childhood adversity, employing truncation symbols to capture variations in word forms and phrases. Additionally, we included terms related to child behaviour and socio‐emotional development. To ensure the inclusion of studies examining potential mechanisms of transmission, we further incorporated terms referring to statistical mediation. The different terms were combined using Boolean operators ‘OR’ and ‘AND’ to structure the search and maximise the identification of relevant studies (see Supporting Information for the search strings). The searches ran from inception to February 16, 2026.

### 2.2 Inclusion and Exclusion Criteria

All peer‐reviewed observational studies evaluating mechanisms in the association between maternal ACEs and child socio‐emotional/behavioural outcomes were considered, including retrospective and prospective cohort, case–control and cross‐sectional studies. There was no language restriction. Qualitative studies, review studies, grey literature, opinion articles, commentaries and editorials were excluded.

We focused on the biological, psychological and social mechanisms that occur during pregnancy. Studies evaluating mechanisms occurring from birth onwards (postnatal mediators) were excluded if there was no separate analysis to identify the effect of prenatal mediators. When studies did not report the mediator's effect size, the corresponding author was contacted to request additional information.

We included children aged 0–18 years whose outcome measures were assessed before 18 years of age. Outcomes included: internalising symptoms (e.g., anxiety, depression, withdrawal, somatisation), externalising symptoms (e.g., hyperactivity/inattention, aggression/defiance, impulsivity), temperament measures, and any diagnosed mental disorder. We excluded children whose socio‐emotional development was not assessed or studies that assessed global child development without a separate analysis for socio‐emotional or behavioural development.

Any measure of ACEs from the mother's childhood (before Age 18) was considered an exposure variable. Studies that assess experiences outside the family setting, such as witnessing violence in the community or racial discrimination, were also included [22]. Studies in which adverse events occurred after the mother's 18th birthday were excluded.

### 2.3 Screening

Rayyan software [23] was used to eliminate duplicates and to perform the initial selection of titles and abstracts. The first step consisted of title and abstract screening, which was performed using the database searches according to the inclusion and exclusion criteria. This screening process was carried out independently by two different reviewers (S.A. and A.M.). A third reviewer (J.W.) screened 10% of the titles and abstracts and was consulted for consensus when necessary. In the second phase, full texts were retrieved for detailed evaluation. Studies that met all inclusion criteria were selected. Disagreements were discussed and resolved by consensus between the reviewers. Zotero was used for citation management of the included articles.

### 2.4 Data Extraction

Data extraction was performed by two independent reviewers (S.A. and A.M.) using a standardised extraction form. The following items were collected from the retained studies: (i) characteristics of the study: author, year, country of origin; (ii) study design, sample size, study setting (e.g., clinical vs. community setting); (iii) maternal ACEs (measures used and type of ACEs); (iv) child outcomes; (v) child age at outcome measurement; (vi) mediators (measures used, direction of association and effect size). Any differences between the two reviewers (S.A. and A.M.) were discussed with JW to reach consensus, if needed.

### 2.5 Quality Assessment

Methodological quality of the studies was assessed using the adapted version of the Newcastle‐Ottawa Scale (NOS) for cohort, cross‐sectional and case–control studies [24]. The NOS evaluates bias in nonrandomized studies across three domains: participant selection, comparability and ascertainment of the outcome variable. We modified the item ‘ascertainment of exposure’ to award 1 point when a validated instrument was used to measure maternal ACEs and 0 points when the ACEs instrument was not validated (Table 1). Each study was assigned an overall score ranging from 0 to 9 points, with ≥ 7 indicating a good‐quality study, 5–6 a fair‐quality study and ≤ 4 a poor‐quality study [8].

**TABLE 1:** Quality assessment criteria based on the adapted Newcastle‐Ottawa scale for cohort, cross‐sectional and case–control studies.

Criterion	Cohort studies	Cross‐sectional studies	Case–control studies
Selection	Representativeness of the exposed cohort ★ Truly representative; ★ Somewhat representative; (—) Selected group; (—) No description	Representativeness of the cases ★ Truly representative; ★ Somewhat representative; (—) Selected group; (—) No description	Adequate case definition ★ Yes; (—) No
	Selection of the non‐exposed cohort ★ Drawn from the same community; (—) No description	Non‐response rate ★ Low response rate with clear description; (—) No description	Representativeness of the cases ★ Consecutive or obviously representative; (—) Selected from records; (—) No description
	Ascertainment of exposure ★ Validated tool; (—) Non‐validated tool	Ascertainment of exposure ★ Validated tool; (—) Non‐validated tool	Selection of controls ★ Community controls; (—) Hospital controls; (—) No description
	Demonstration that outcome was not present at start ★ Yes; (—) No	Not applicable	Not applicable
Comparability	Control for main confounding factors^a^ ★ Yes; (—) No	Control for main confounding factors^a^ ★ Yes; (—) No	Control for main confounding factors^a^ ★ Yes; (—) No
	Control for additional factors^b^ ★ Yes; (—) No	Control for any additional factors^b^ ★ Yes; (—) No	Control for any additional factors^b^ ★ Yes; (—) No
Outcome	Assessment of outcome ★ Independent blind assessment or record linkage; ★ Self‐report; (—) No description	Assessment of outcome ★ Independent blind assessment or record linkage; ★ Self‐report; (—) No description	Ascertainment of exposure ★ Secure record or structured interview; ★ Self‐report; (—) No description
	Adequacy of follow‐up ★ Complete follow‐up; ★ Some loss to follow‐up; (—) No statement	Statistical test appropriateness ★ Appropriate statistical test used; (—) Inappropriate test; (—) No description	Same method of ascertainment for cases and controls ★ Yes; (—) No Non‐response rate ★ Same for both groups; (—) Different between groups; (—) No description

*Note:* In cursive, the adaptations made to the quality assessment instrument for the current study. Each domain is scored as either met (★, 1 point) or not met (—, 0 points).

^a^ Socioeconomic status (SES), or some reasonable proxy of SES like age, race, gender.

^b^ These criteria were modified to indicate specific control for additional important factors.

### 2.6 Data Synthesis

To support the narrative synthesis of our findings, we compiled a table (Table 2) summarising the mediating effects of prenatal mechanisms and comparing key elements across studies including mediation effect sizes, consideration of postnatal factors, and stratification by the child's biological sex. Results were then collaboratively reviewed and discussed to identify the most reported prenatal mechanisms linking maternal ACEs to child socio‐emotional development.

**TABLE 2:** Mediating effects of prenatal mechanisms identified in included studies (*n* = 13).

Authors (reference numbers)	Mediators (instrument used)	Tested mediating pathway X → M → Y	Indirect effect via prenatal mediator	Postnatal factors	Accounting for child sex
Ahlfs‐Dunn et al. [25]	Disrupted prenatal maternal representations of the child (WMCI)	↑ Maternal childhood interpersonal trauma history → ↑ disrupted representation of child → ↑ infant socio emotional problems	*β* = 0.26^a^	—	—
Isosävi et al. [26]	PTSD (31‐item Harvard Trauma Questionnaire), Depression symptoms (EPDS), Perceived stress (PSS)	↑ Maternal childhood emotional abuse → ↑ prenatal depressive symptoms → ↑ soothability	*β* = 0.04^a^	—	As confounder
Khoury et al. [27]	Maternal prenatal depression symptoms (CES‐D)	↑ Maternal ACEs score → ↑ prenatal depression symptoms → ↑ child socioemotional problems (intercept at 15 months old)	*β* = 0.14 (95% CI 0.05, 0.03)^b^	Prenatal depression independently mediated the link between maternal ACEs and child outcomes even when accounting for maternal postnatal depression Serial indirect effect through prenatal and postnatal depression: *β* = 0.03, 95% CI −0.01, 0.08	As confounder
	Maternal prenatal depression symptoms (CES‐D)	↑ Maternal ACEs score → ↑ prenatal depression symptoms → ↑ child socioemotional problems (slope representing change in child symptoms from 15, 24, 35 months old)	*β* = −0.002 (95% CI −0.06, 0.05)	Serial indirect effect through prenatal and postnatal depression: *β* = 0.02, 95% CI −0.01, 0.05	As confounder
Khoury et al. [28]	Maternal prenatal mental health symptoms: depressive (EPDS), anxiety (GAD‐7), perceived stress (PSS), borderline personality (BSL‐23)	↑ Maternal childhood maltreatment → ↑ prenatal mental health symptoms → ↑ infant socioemotional problems	*β* = −0.07 (95% CI −0.19, 0.05)	Serial indirect effect through prenatal and postnatal mental health symptoms: *β* = 0.105, 95% CI 0.019, 0.242	—
	Maternal prenatal depressive symptoms (EPDS)	↑ Maternal childhood maltreatment → ↑ prenatal depressive symptoms → ↑ infant socioemotional problems	*β* = −0.04 (95% CI −0.13, 0.03)	Serial indirect effect through prenatal and postnatal depression symptoms: *β* = 0.074 95% CI 0.010, 0.144	—
	Maternal prenatal anxiety symptoms (GAD)	↑ Maternal childhood maltreatment → ↑ prenatal anxiety symptoms → ↑ infant socioemotional problems	*β* = −0.04 (95% CI [−0.13, 0.03])	Serial indirect effect through prenatal and postnatal anxiety symptoms: *β* = 0.070, 95% CI 0.016, 0.149	—
	Maternal prenatal perceived stress symptoms (PSS)	↑ Maternal childhood maltreatment → ↑ prenatal perceived stress symptoms → ↑ infant socioemotional problems	*β* = −0.03 (95% CI −0.13, 0.06)	Serial indirect effect through prenatal and postnatal perceived stress symptoms: *β* = 0.029, 95% CI −0.015, 0.084	—
	Maternal prenatal borderline personality symptoms (BSL)	↑ Maternal childhood maltreatment → ↑ prenatal borderline personality symptoms → ↑ infant socioemotional problems	*β* = −0.04 (95% CI −0.15, 0.05)	Serial indirect effect through prenatal and postnatal borderline personality symptoms: *β* = 0.032, 95% CI 0.002, 0.087	—
Letourneau et al. [29]	Maternal prenatal depression (EPDS) and anxiety (Symptoms Checklist‐90‐Revised Questionnaire)	↑ Maternal ACEs score → ↑ prenatal anxiety → ↑ child externalising symptoms	*β* = 0.026 (95% CI −0.010, 0.085)^b^	—	Boys: (−0.01, 0.16)^c^ Girls: (−0.01, 0.10)^c^
		↑ Maternal ACEs score → ↑ prenatal depression → ↑ child externalising symptoms	*β* = 0.02 (95% CI −0.001, 0.06)^b^	—	Boys: (−0.02, 0.06)^c^ Girls: (−0.01, 0.06)^c^
		↑ Maternal ACEs score → ↑ prenatal depression and anxiety → ↑ child externalising symptoms	95% CI 0.02–0.09^c^	—	Boys: 95% CI (0.02, 0.14)^c^ Girls: (−0.003, 0.099)^c^
		↑ Maternal ACEs score → ↑ prenatal anxiety → ↑ child internalising symptoms	*β* = 0.07 (95% CI 0.02, 0.14)^b^	Post hoc analyses: antenatal anxiety mediating effects disappeared when postnatal depression was considered	Boys: (0.035, 0.265)^c^ Girls: (−0.001, 0.140)^c^
		↑ Maternal ACEs score → ↑ prenatal depression → ↓ child internalising symptoms	*β* = −0.004, 95% CI −0.03, 0.02^b^	—	Boys: (−0.079, 0.025)^c^ Girls: (−0.036, 0.029)^c^
		↑ Maternal ACEs score → ↑ prenatal depression and anxiety → ↑ child internalising symptoms	95% CI 0.03–0.12^c^	—	Boys: (0.045, 0.209)^c^ Girls: (0.002, 0.111)^c^
Mattera et al. [30]	Internalising symptoms: Maternal prenatal depression (EPDS) and anxiety (STAI)	↑ Maternal ACEs → ↑ prenatal internalising symptoms → ↓ Falling reactivity	*β* = −0.18, 95% CI −0.35, −0.03^b^	Maternal sensitivity/responsiveness at 2 months postpartum This indirect effect of internalising symptoms was present at low (*β* = −0.15, 95% CI −0.64, −0.001)^b^ and moderate (*β* = −0.16, 95% CI −0.40, −0.03)^b^ but not high levels of maternal sensitivity/responsiveness (*β* = −0.17, 95% CI −0.41, 0.008)^b^	Only as confounder
		↑ Maternal ACEs → ↑ prenatal depression symptoms → ↓ Falling reactivity	*β* = −0.21, 95% CI −0.39, −0.05^b^	Maternal sensitivity/responsiveness at 2 months postpartum The indirect effect of depression was present at low (*β* = −0.18, 95% CI −0.521, −0.01)^b^ and moderate (*β* = −0.17, 95% CI −0.37, −0.02)^b^ but not high levels of maternal sensitivity/responsiveness (*β* = −0.15, 95% CI −0.38, 0.04)^b^	
		↑ Maternal ACEs → ↑ prenatal internalising symptoms → ↑ soothability	*β* = 0.12^a^	—	
		↑ Maternal ACEs → ↑ prenatal depression symptoms → ↓ soothability	*β* = −0.14^a^	—	
		↑ Maternal ACEs → ↑ prenatal anxiety symptoms → ↓ soothability	*β* = −0.11^a^	—	
Min et al. [31]	Prenatal substance exposure (alcohol, tobacco, marijuana, cocaine)	↑ Maternal childhood maltreatment → ↑ prenatal substance exposure → ↑ child internalising and externalising symptoms	There was no association between maternal ACEs and prenatal substance use, therefore, the mediating pathway was not reported		
Moon et al. [32]	Antepartum health risks (obstetric evaluation tool)	↑ Maternal ACEs score → ↑ antepartum health risks → ↑ ADHD symptoms	*β* = 0.04 (95% CI 0.01, 0.12)^b^	—	—
Plant et al. [33]	Maternal prenatal depression (EPDS)	↑ Maternal childhood maltreatment → ↑ prenatal depression → ↑ child internalising symptoms	*β* = 0.01 (95% CI 0.01, 0.02)^b^	Antenatal depression independently mediated the link between maternal childhood maltreatment and child outcomes even when accounting for maternal postnatal depression, child maltreatment	Only as confounder
		↑ Maternal childhood maltreatment → ↑ antenatal depression → ↑ child externalising symptoms	*β* = 0.009 (95% CI 0.005, 0.012)^b^		
Roberts et al. [34]	Perinatal health risks	Maternal childhood abuse → gestational diabetes → child autism	Mediated proportion of 3%^d^	—	Only as confounder
		Maternal childhood abuse → smoking during pregnancy → child autism	Mediated proportion of 2%^d^	—	
Roth et al. [35]	Maternal fetal attachment (ASQ)	↑ Maternal ACEs → ↓ secure attachment domain → ↑ child emotional and behavioural problems	*β* = 0.09 (95% CI 0.003, 0.21)^b^	—	Only as confounder
		↑ Maternal ACEs → ↑ anxious attachment domain → ↑ child emotional and behavioural problems	*β* = 0.07 (95% CI −0.03, 0.19)^b^	—	
		↑ Maternal ACEs → ↑ avoidant attachment domain → ↑ child emotional and behavioural problems	*β* = 0.04 (95% CI −0.04, 0.18)^b^	—	
Thomas‐Argyriou et al. [36]	Prenatal maternal HPA‐axis function (cortisol awakening response (CAR), Diurnal slope, AUCg)	↑ Maternal ACEs → ↑ maternal CAR at T1 (6–26 weeks gestation) → ↓ child internalising symptoms	*β* = −0.21 (95% CI −0.53, −0.04)^b^	Results were adjusted for maternal postnatal distress and postnatal stressful life events	Only as confounder: Adding child sex to the model did not yield any further findings
		↑ Maternal ACEs → ↑ maternal CAR at T2 (27–37 weeks gestation) → ↓ child internalising symptoms	*β* = −0.14 (95% CI −0.48, −0.002)^b^		
Uy et al. [37]	Maternal prenatal depression (BDI‐2) and anxiety (STAI)	↑ Maternal childhood emotional neglect → → ↑ prenatal anxiety and depressive symptoms → ↑ child internalising problems	*β* = 0.06 (95% CI −0.01, 0.15)^b^	Serial indirect effect through prenatal and postnatal mental health: *β* = 0.07 (95% CI 0.02, 0.12)^b^	Only as confounder
		↑ Maternal childhood physical neglect → ↑ prenatal anxiety and depressive symptoms → ↑ child internalising problems	*β* = 0.12 (95% CI −0.03, 0.23)^b^	Serial indirect effect through prenatal and postnatal mental health: *β* = 0.14 (95% CI 0.06, 0.25)^b^	
		↑ Maternal childhood emotional abuse → ↑ prenatal anxiety and depressive symptoms → ↑ child internalising problems	*β* = 0.37 (95% CI −0.11, 0.93)^b^	Serial indirect effect through prenatal and postnatal mental health: *β* = 0.42 (95% CI 0.08, 0.94)^b^	
		↑ Maternal childhood physical abuse → ↑ prenatal anxiety and depressive symptoms → ↑ child internalising problems	*β* = 0.29 (95% CI −0.09, 0.86)^b^	Serial indirect effect through prenatal and postnatal mental health: *β* = 0.34 (95% CI 0.01, 0.86)^b^	
		↑ Maternal childhood sexual abuse → ↑ prenatal anxiety and depressive symptoms → ↑ child internalising problems	*β* = 0.05 (95% CI −0.26, 0.43)^b^	Serial indirect effect through prenatal and postnatal mental health: *β* = 0.07 (95% CI −0.25, 0.48)^b^	
		↑ Maternal childhood total neglect → ↑ prenatal anxiety and depressive symptoms → ↑ child total anxiety	*β* = 0.14 (99.2% CI −0.08, 0.38)^b^	Postnatal mental health did not mediate the association between maternal childhood trauma and child self‐reported anxiety/depression	
		↑ Maternal childhood total abuse → ↑ prenatal anxiety and depressive symptoms → ↑ child total anxiety	*β* = 0.38 (99.2% CI −0.34, 1.23)^b^		
		↑ Maternal childhood total neglect → ↑ prenatal anxiety and depressive symptoms → ↓ child total depression	*β* = −0.07 (99.2% CI −0.35, 0.17)^b^		
		↑ Maternal childhood total abuse → ↑ prenatal anxiety and depressive symptoms → ↓ child total depression	*β* = −0.14 (99.2% CI −1.09, 0.57)^b^		

Abbreviations: ASQ, Attachment Style Questionnaire; AUCg, Area Under the Curve; BDI‐2, Beck Depression Inventory‐II; BSL‐23, Borderline Symptom List‐23; CAR, Cortisol Awakening Response; CES‐D, Center for Epidemiologic Studies Depression Scale; EPDS, Edinburgh Postnatal Depression Scale; GAD‐7, Generalised Anxiety Disorder‐7; PSS, Perceived Stress Scale; STAI, State–Trait Anxiety Inventory; WMCI, Working Model of the Child Interview.

^a^ Only reported *β*.

^b^ Reported as *β* [95% CI].

^c^ Reported 95% CI only, no point estimate.

^d^ Reported as mediated proportion, not *β*.

### 2.7 Ethics Approval

Ethics approval was not required for this study as it is a systematic review of previously published literature and did not involve primary data collection or human subjects.

## 3 Results

### 3.1 Included Studies

The electronic search yielded 2641 studies; 122 were selected for full‐text screening, and 13 met full inclusion criteria (Figure 1). Most studies employed a longitudinal cohort design and were conducted in North America (*n* = 9) in community settings. However, three studies focused on at‐risk populations [25, 26, 31] including women living in war zones, economically disadvantaged samples, and a convenience sample [35] by recruitment of pregnant women attending a psychiatric clinic. Nine studies were published after 2020. Table 3 provides detailed characteristics of included studies (study design, sample characteristics, ACE measurement and outcome measures). Table S1 provides an overview of the criteria that led to article exclusion during the full‐text screening process.

**FIGURE 1:**
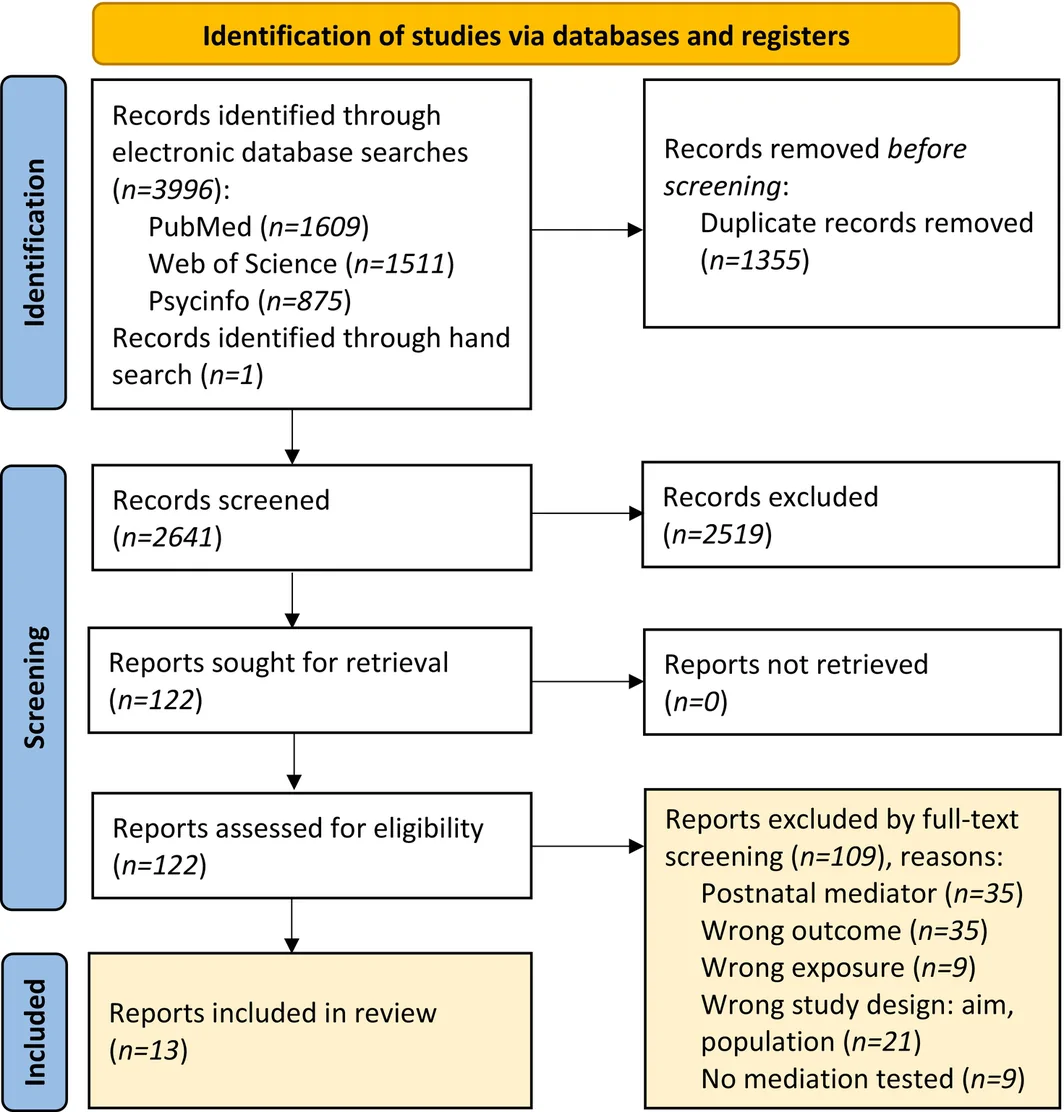
PRISMA 2020 flow diagram for new systematic reviews which included searches of databases.

**TABLE 3:** Summary of characteristics of included studies (*n* = 13).

Authors (reference number)	Study title	Year	Country	Study design	Sample size	Sample recruitment	ACEs type (instrument used)	Child age (average)	Child outcomes (instrument used)	Quality assessment
Ahlfs‐Dunn et al. [25]	Intergenerational transmission of trauma from mother to infant: the mediating role of disrupted prenatal maternal representations of the child	2021	USA	Cohort study	120	High‐risk population (sample predominantly composed of single and economically disadvantaged women)	Childhood abuse and neglect (CTQ) Intimate partner violence during childhood (ROME‐M)	12 months	Internalising and externalising symptoms (BITSEA)	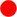
Isosävi et al. [26]	Maternal trauma affects prenatal mental health and infant stress regulation among Palestinian dyads	2017	Palestine	Cohort study	511	High‐risk population (women living in the Gaza Strip)	Childhood emotional abuse, childhood physical abuse, childhood neglect (13 item questionnaire from the Transcultural Psychosocial Organisation)	4 months	Infant stress regulation (IBQ‐R)	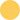
Khoury et al. [27]	Adverse Childhood Experiences, Pregnancy and Postpartum Depressive Symptoms, and Child Socioemotional Problems Over the First Three Years of Life	2025	Canada	Cohort study	304	Community setting	Overall ACEs score (10 item ACEs questionnaire by Felitti et al.)	15‐, 24‐, and 35‐months	Infant socio‐emotional problems (BITSEA‐Problem scale)	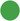
Khoury et al. [28]	Linking maternal childhood maltreatment to infant socioemotional development through perinatal mental health	2026	Canada	Cohort study	128	Community setting	Childhood abuse and neglect (CTQ‐SF)	6 months	Infant socio‐emotional problems (ASQ:SE‐2)	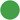
Letourneau et al. [29]	Intergenerational transmission of adverse childhood experiences via maternal depression and anxiety and moderation by child sex	2018	Canada	Cohort study	907	Community setting	Childhood maltreatment + household dysfunction (10 item ACEs questionnaire by Felitti et al.)	24 months	Internalising and externalising symptoms (CBCL)	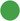
Mattera et al. [30]	Prenatal internalising symptoms as a mediator linking maternal adverse childhood experiences with infant temperament	2022	USA	Cohort study	64	Community setting	Overall ACEs score (10 item ACEs questionnaire by Felitti et al.)	2 months	Infant temperament (IBQ‐R)	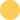
Min et al. [31]	Intergenerational transmission of maternal childhood maltreatment, prenatal substance exposure, and internalising and externalising symptoms in early adolescence at Age 12	2026	USA	Cohort study	284	High‐risk population (low‐income adolescents—half of their mothers used substances during pregnancy)	Childhood maltreatment (CTQ)	12 years	Internalising and externalising symptoms (CBCL; YSR)	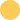
Moon et al. [32]	Association between maternal adverse childhood experiences and attention‐deficit/hyperactivity disorder in the offspring: the mediating role of antepartum health risks	2021	South Korea	Cross‐sectional	461	Community setting	Overall ACEs score (10 item ACEs questionnaire by Felitti et al.)	13 years	ADHD symptoms (DPS)	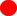
Plant et al. [33]	Association between maternal childhood trauma and offspring childhood psychopathology: mediation analysis from the {ALSPAC} cohort	2017	UK	Cohort study	9397	Community setting	Childhood maltreatment score (not validated questionnaire)	10–13 years	Internalising and externalising symptoms (DAWBA and SDQ)	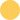
Roberts et al. [34]	Maternal exposure to childhood abuse is associated with elevated risk of autism	2013	USA	Case–control study	52,949	Community setting	Childhood maltreatment abuse (CTQ) Childhood sexual abuse	5 years	Autism (Autism Diagnostic Interview‐Revised)	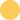
Roth et al. [35]	Attachment security in pregnancy mediates the association between maternal childhood maltreatment and emotional and behavioural problems in offspring	2021	USA	Cohort study	124	Convenience sample (pregnant women attending/being treated in a psychiatric clinic)	Childhood maltreatment (CTQ)	4.4 years	Internalising and externalising symptoms (CBCL)	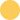
Thomas‐Argyriou et al. [36]	The role of HPA‐axis function during pregnancy in the intergenerational transmission of maternal adverse childhood experiences to child behaviour problems	2020	Canada	Cohort study	248	Community setting	Overall ACEs score (10 item ACEs questionnaire by Felitti et al.)	48 months	Internalising and externalising symptoms (BASC‐2)	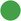
Uy et al. [37]	Effects of maternal childhood trauma on child emotional health: maternal mental health and frontoamygdala pathways	2023	Singapore	Cohort study	541	Community setting	Maternal childhood abuse score (CTQ) Maternal childhood neglect score (CTQ)	7.5 years	Internalising problems (CBCL), depression symptoms (CDI) and anxiety symptoms (MASC‐2)	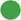

*Note:* The overall quality rating (far right) is indicated by coloured circles:  green (≥ 7 points = good quality),  yellow (5–6 points = fair quality),  red (≤ 4 points = poor quality). Reference numbers for each study are provided in parentheses.

Abbreviations: ADI‐R, Autism Diagnostic Interview‐Revised; ASQ:SE‐2, Ages and Stages Questionnaires: Social–Emotional (Second Edition); BASC‐2, Behaviour Assessment System for Children‐2; BITSEA, Brief Infant‐Toddler Social and Emotional Assessment; CBCL, Child Behaviour Checklist; CDI, Children's Depression Inventory; CTQ, Childhood Trauma Questionnaire; CTQ‐SF, CTQ Short Form; DAWBA, Development and Well‐Being Assessment; DPS, Diagnostic Predictive Scales; IBQ‐R, Infant Behaviour Questionnaire‐Revised; MASC‐2, Multidimensional Anxiety Scale for Children‐2; ROME‐M, Revised Conflict Tactics Scale; SDQ, Strengths and Difficulties Questionnaire; YSR, Youth Self‐Report.

Participants included 66,038 mother–child dyads. Mothers averaged 30.2 years old at inclusion; children were assessed at a mean age of 45.8 months (range: 2 months–13 years), with approximately 48% of the sample being boys. Maternal ACEs were most often measured with the 10‐item ACEs questionnaire [1] (*n* = 5) and the Childhood Trauma Questionnaire [38] (*n* = 6). Eight studies [27, 28, 29, 30, 32, 33, 35, 36] calculated an overall ACEs score, with 16% of mothers reporting three or more ACEs, whereas two studies [25, 31] employed a latent construct of childhood maltreatment and trauma history using structural equation modelling.

Regarding child outcomes, infant temperament (Ages 2–4 months) was measured using the parent‐reported Infant Behaviour Questionnaire‐Revised (IBQ‐R) [39] (*n* = 2) [26, 30]. Internalising and externalising symptoms were evaluated in infants (Ages 12–35 months) with the Brief Infant‐Toddler Social and Emotional Assessment (BITSEA) [40] (*n* = 2) [25, 27] and the Ages and Stages Questionnaires: Social–Emotional (ASQ:SE) [41] (*n* = 1) [28]. In older children and adolescents (Ages 4–18 years), these symptoms were measured with multiple instruments including the Child Behaviour Checklist (CBCL) [42] (*n* = 4) [29, 31, 35, 37], Development and Well‐Being Assessment (DAWBA) [43] (*n* = 1) [33] and Strengths and Difficulties Questionnaire (SDQ) [44] (*n* = 1) [33], among other validated instruments.

### 3.2 Quality Assessment

Based on the adapted NOS, five studies had good methodological quality, six had fair quality and two had poor quality (Table 4). A key limitation across studies was the reliance on parent‐reported measures to assess child outcomes. Additionally, most studies lacked adequate description or comparison of unexposed groups.

**TABLE 4:** Quality assessment for included studies using an adapted version of the Newcastle‐Ottawa scale.

Author, year (reference numbers)	Study design	Selection				Comparability		Outcome			Overall
		Representativeness of the exposed cohort	Selection of the non‐exposed cohort	Ascertainment of exposure	Demonstration that outcome of interest was not present at start of study	Study controls for the most important factors	Study control for any additional factor	Assessment of outcome	Was follow‐up long enough for outcomes to occur	Adequacy of follow up of cohorts	
Ahlfs‐Dunn et al., 2021 [25]	Cohort	—	—	★	★	—	—	—	★	★	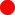
Isosävi et al., 2017 [26]	Cohort	—	—	—	★	★	★	—	★	★	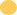
Khoury et al., 2025 [27]	Cohort	★	★	★	★	★	★	—	★	★	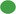
Khoury et al., 2026 [28]	Cohort	★	—	★	★	★	★	—	★	★	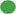
Letourneau et al., 2018 [29]	Cohort	★	—	★	★	★	★	—	★	★	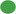
Mattera et al., 2022 [30]	Cohort	★	—	★	★	★	—	—	★	★	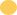
Min et al., 2026 [31]	Cohort	—	—	★	★	★	★	—	★	★	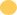
Plant et al., 2017 [33]	Cohort	★	★	—	★	★	★	—	★	—	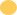
Roth et al., 2021 [35]	Cohort	—	—	★	★	★	★	—	★	—	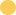
Thomas‐Argyriou et al., 2020 [36]	Cohort	★	—	★	★	★	★	—	★	★	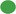
Uy et al., 2023 [37]	Cohort	★	—	★	★	★	★	—	★	★	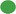
		**Representativeness of the cases**	**Nonresponse rate**	**Ascertainment of exposure**		**Study controls for the most important factors**	**Study control for any additional factor**	**Assessment of outcome**	**Statistical test**		**Overall**
Moon et al., 2021 [32]	Cross‐sectional	—	—	★		★	★	—	★		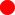
		**Adequate case definition**	**Representativeness of the cases**	**Selection of controls**	**Definition of control**	**Study controls for the most important factors**	**Study control for any additional factor**	**Assessment of exposure**	**Same method of ascertainment for cases and controls**	**Non‐ response rate**	**Overall**
Roberts et al., 2013 [34]	Case–control	—	★	★	★	★	—	★	★	—	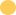

*Note:* Study quality was assessed using an adapted version of the Newcastle‐Ottawa Scale (NOS), with criteria modified according to study design (cohort, cross‐sectional, or case–control). Each domain is scored as either met (★, 1 point) or not met (—, 0 points). The overall quality rating (far right) is indicated by coloured circles:  green (≥ 7 points = good quality),  yellow (5–6 points = fair quality),  red (≤ 4 points = poor quality). Reference numbers for each study are provided in parentheses.

### 3.3 Mediating Mechanisms Occurring in Pregnancy

Nine studies [25, 26, 27, 28, 29, 30, 33, 35, 37] evaluated psychological mediators including prenatal depression and anxiety, borderline personality symptoms, perceived stress, maternal attachment and maternal representations of the child. Two studies [32, 34] evaluated antepartum health risks, and one study [31] examined the role of prenatal substance use. One study [36] explored biological markers, focusing in particular on hypothalamic–pituitary–adrenal (HPA) axis function during pregnancy. See Table 2 and Figure 2 for a summary of the identified prenatal mediating pathways.

**FIGURE 2:**
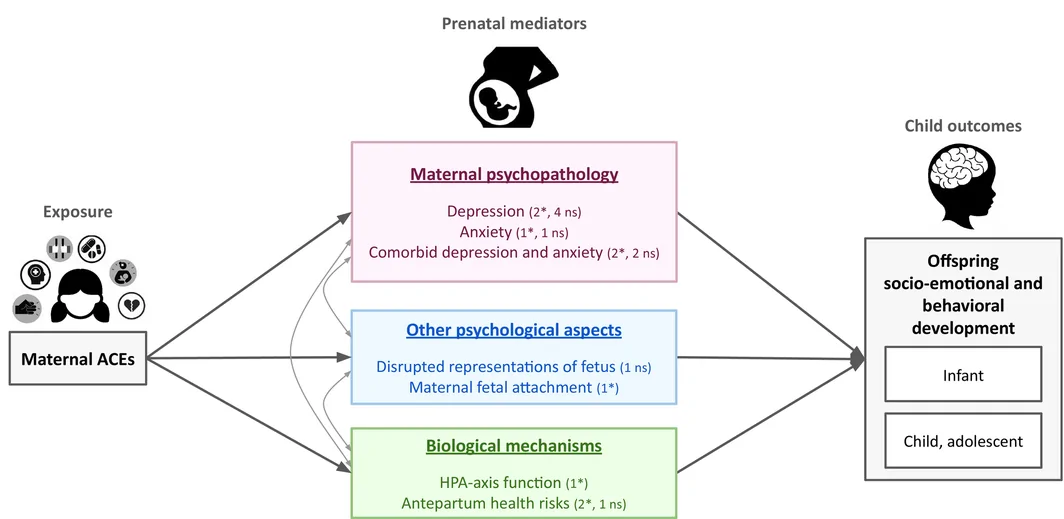
Mediation pathways linking maternal ACEs to offspring outcomes through prenatal mechanisms. The figure depicts three categories of prenatal mediators (boxes in the middle) identified in this systematic review: Maternal psychopathology (light pink), other psychological aspects (light blue), and biological mechanisms (light green). Solid black arrows indicate pathways examined by included studies. Grey curved arrows between mediator categories represent potential interconnections among these mechanisms. For each mediator, in parentheses, we show the number of studies finding significant mediation effects (*) vs. non‐significant effects (ns).

#### 3.3.1 Prenatal Maternal Depression

Six studies [26, 27, 28, 29, 30, 33] evaluated prenatal depression as a mediator. Three studies [26, 28, 29] found no mediating effect on child outcomes including infant temperament and stress regulation, and externalising or internalising symptoms at Age 2. In contrast, two studies [27, 33] found indirect effects of maternal ACEs on child outcomes via prenatal depression, with effects persisting independently of postnatal factors. Particularly, Mattera et al. [30] found an indirect effect linking a maternal ACEs score with infant falling reactivity via prenatal depression symptoms, only present at low‐moderate levels of maternal sensitivity in the postpartum.

#### 3.3.2 Prenatal Maternal Anxiety

Two studies [28, 29] evaluated the effect of a maternal ACEs score on child outcomes via prenatal anxious symptoms. One study [29] found that prenatal anxious symptoms had a mediating effect in the relationship between maternal ACEs score and child internalising symptoms, whereas the other study [28] did not find a mediating effect of prenatal anxiety.

#### 3.3.3 Combined Prenatal Mental Health Difficulties

Four studies [28, 29, 30, 37] analysed whether concomitant depression and anxiety during pregnancy were mediators between maternal ACEs and child outcomes. Two of them found mediating effects on child externalising and internalising symptoms [29, 37], whereas the other two showed mediating effects under certain postnatal conditions, such as the serial mediation effect of both prenatal and postnatal symptoms [28], and moderation by postnatal maternal sensitivity, with the mediating effect being evident only at low and moderate levels of sensitivity [30]. Post hoc analyses performed by Letourneau et al. [29] indicated that maternal ACEs had an indirect effect on child externalising symptoms via prenatal depression and anxiety when modelled separately, but neither showed a unique effect when modelled together, suggesting considerable overlap in their effects.

#### 3.3.4 Maternal–Fetal Attachment

Roth et al. [35] explored the mediating role of maternal attachment style (secure, anxious and avoidant dimensions) in the third trimester of pregnancy. They found that maternal ACEs predicted lower secure attachment, leading to higher child emotional/behavioural problems. The anxious and avoidant dimensions didn't show a mediating effect.

#### 3.3.5 Disrupted Prenatal Maternal Representations of the Child

Ahlfs‐Dunn et al. [25] evaluated the role of maternal representations of the child, defined as a mother's internalised thoughts, emotions, and expectations regarding their child and their maternal role. Although maternal childhood trauma was associated with disrupted prenatal representations (defined as contradictory or incoherent narratives), these representations were not linked to child socio‐emotional difficulties.

#### 3.3.6 Antepartum Health Risks and Substance Use

Two studies [32, 34] examined the role of antepartum health risks as mediators. Moon et al. [32] found an indirect association between maternal ACEs and child's ADHD symptoms via an overall antepartum risk score. In a case–control study, Roberts et al. [34] reported that maternal childhood abuse was associated with an increased offspring autism risk, partially mediated by gestational diabetes and prenatal smoking. Furthermore, Min et al. [31] found that prenatal substance use (alcohol, tobacco, marijuana and cocaine) was not in the pathway from maternal childhood maltreatment to offspring's psychopathology symptoms in adolescence.

#### 3.3.7 HPA‐Axis Function During Pregnancy

Only one study [36] assessed biomarkers, focusing on the maternal cortisol awakening response (CAR), measured at 2 time points. Increased CAR at both time points, linked to maternal ACEs, was associated with fewer offspring internalising symptoms at 4 years. These findings suggest that prenatal CAR may function as a potential resilience factor under moderate prenatal stress.

Total cortisol levels, diurnal slope and area under the curve (AUC) did not show mediating effects on child outcomes.

### 3.4 Effect Modification by Child Sex

Only one study [29] explored the effect modification by child sex. The model supported a mediator effect of combined prenatal anxiety and depression on externalising symptoms for boys only. In addition, there were combined indirect effects of prenatal anxiety and depression on internalising behaviour for both boys and girls.

## 4 Comment

### 4.1 Principal Findings

This systematic review aimed to identify prenatal mechanisms contributing to the association between maternal ACEs and child socio‐emotional and behavioural outcomes. Despite growing interest in Barker's DoHaD framework [10], these mechanisms remain underexplored. Our review identified 13 studies, predominantly focusing on maternal psychopathology; few examined other psychological aspects (maternal–fetal attachment, prenatal representations of the child) or biomedical mediators (antepartum health risks, HPA‐axis function). Overall, prenatal depression and anxiety appear to contribute to the intergenerational effects of ACEs, not only through their interaction with the postnatal environment, but also as independent mediators.

### 4.2 Strengths of the Study

Most of the studies included in our review utilised prospective longitudinal designs, following mother–child dyads from pregnancy through infancy, childhood, or even pre‐adolescence. In addition, several studies identified modifiable prenatal risk factors (e.g., maternal depression, anxiety and biomedical pregnancy risks) with clinical relevance.

### 4.3 Limitations of the Data

Nonetheless, our work has certain limitations. First, maternal ACEs were assessed via retrospective self‐report, with some studies using unvalidated tools, although such measures have demonstrated predictive validity [45], and this was considered in our adapted quality assessment. Second, the small number of eligible studies (*n* = 13) underscores the limited exploration of prenatal transmission pathways, reflecting the challenges of recruiting and following pregnant populations. Our focus on socio‐emotional and behavioural outcomes led us to exclude studies addressing global child development [46, 47], those combining prenatal and postnatal mediators [48, 49, 50], and those not distinguishing maternal childhood from adulthood trauma [51], suggesting a potentially larger literature base. We updated our search to ensure the review remains current; however, one database (PsycINFO) was inaccessible during the final update. Given that relevant articles in this field are typically indexed across multiple databases, we expect the impact on our findings to be limited, though we acknowledge this as a potential limitation. Third, heterogeneity in ACEs definitions, timing of outcome assessment and analytical strategies made it difficult to synthesise results quantitatively. Fourth, only one study [29] used sex‐stratified analyses, yet the exposure to maternal ACEs may differentially affect socio‐emotional/behavioural development by child biological sex [52]. Finally, the overall study quality was fair. Future research would benefit from the use of validated ACEs assessment tools, comparisons between exposed and unexposed groups, and strategies to address attrition.

### 4.4 Interpretation

#### 4.4.1 Maternal Psychopathology During Pregnancy

Several mechanisms may explain the prenatal mediation effects of depression and anxiety independent of postnatal factors. ACEs may overactivate stress responses [2] with potentially long‐lasting consequences for emotion dysregulation in adulthood and a diminished ability to adjust to major life transitions [18]. Pregnancy represents one such transition, in which unresolved childhood trauma may resurface, representing a salient source of stress [53] and increasing the vulnerability to prenatal psychopathology [18]. In turn, prenatal stress has been associated with a higher risk for child behavioural difficulties, even when adjusting for postnatal distress [54, 55].

On the other hand, the lack of prenatal mediation independent of postnatal factors in several studies [26, 28, 29, 30] could be explained by the children's age when the outcome was assessed. Effects via prenatal psychopathology may not be immediately apparent in early infancy but become detectable as socio‐emotional and behavioural demands increase with age [27]. The differences in methodological strategies (e.g., serial mediation, moderation) among the studies may also account for the varying results. Despite these discrepancies, the current findings suggest that prenatal depression and anxiety may operate, in certain cases, as independent mediating factors rather than just precursors of postnatal difficulties among mothers with a history of ACEs. This underscores the role of the timing of maternal psychopathology in mental health risk transmission and the importance of examination of the type of prenatal psychopathology and the age of child outcome assessment.

#### 4.4.2 Other Maternal Psychological Aspects

ACEs may also impair prenatal attachment to the child [35, 56] through fewer emotional resources to cope with the challenges of becoming a parent and through interaction with maternal mental health. These relationships have been documented in recent studies, with prenatal depressive symptoms acting as mediators between maternal childhood emotional neglect and fetal attachment [57]. Furthermore, prenatal attachment can be an early indicator of difficulties in maternal behaviour after birth, the mother–child relationship and subsequent child well‐being [56]. Despite the evidence being currently too limited to conclude their intergenerational effects, it could represent a valuable target for early prenatal interventions.

#### 4.4.3 Biological Mechanisms During Pregnancy

The scarcity of mediation studies evaluating prenatal biomarkers reflects the complexity of the downstream effects of maternal ACEs on child outcomes. The unexpected protective effect found in this review [36] (fewer internalising symptoms in children) contrasts with other studies in which elevated total maternal cortisol was linked to higher offspring anxiety and emotional reactivity [58]. Clarifying these associations is crucial to establishing the conditions under which prenatal HPA‐axis dysregulation confers risk versus resilience in the context of maternal ACEs [59].

Moreover, expectant mothers with a history of ACEs may be more vulnerable to pregnancy complications (e.g., gestational diabetes, hypertensive disorders, obesity) [16], which may lead to alterations in the intrauterine environment, including inflammation and endocrine dysregulation. Risky health behaviours like smoking and illicit drug use during pregnancy [17] could further compound these pathways and increase the risk of subsequent offspring mental health difficulties [60]. As these factors are potentially modifiable, early detection and management during pregnancy may help interrupt the cycle of intergenerational adversity.

#### 4.4.4 Postnatal Influences and Child Mental Health Outcomes

Maternal mental health, particularly maternal depression, remains the most widely investigated mediator [7, 8]. However, our review extends prior work into the prenatal period, demonstrating that postpartum mental health acts as a key link within a broader perinatal mediation pathway connecting maternal ACEs and child development. Seven studies [27, 28, 29, 30, 33, 36, 37] in this review employed serial mediation across the perinatal period or examined whether postnatal factors moderated the prenatal mediating effect. Particularly, three studies [28, 33, 37] found an indirect effect of maternal ACEs on child outcomes via the serial mediation of prenatal and postnatal maternal psychopathology, highlighting the importance of persistent mental health symptoms across the perinatal period. These findings support the chain‐of‐risk model [15] from the life‐course theory approach in which prenatal exposures may initiate a trajectory towards negative child outcomes, which are ultimately compounded by postnatal factors. This underscores the necessity of understanding these temporal cascades by evaluating biopsychosocial risk factors at different time points through the perinatal period with tools such as serial mediation models, SEM and the structured life‐course modelling approach (SLCMA) [61] to clarify how these exposures interact and unfold over time.

#### 4.4.5 Implications for Research and Clinical Practice

The emerging evidence presented in this review carries important implications for future research and clinical practice. From a research perspective, our review highlights persistent gaps in understanding the distinct and combined roles of prenatal and postnatal factors in later child outcomes. Future longitudinal research should integrate biological pathways (e.g., HPA‐axis function, inflammation, epigenetics) [62] with psychosocial mediators identified in this review, as well as with social support [59] and PTSD [63]. As prenatal mental health difficulties frequently extend into the postnatal period [64], supporting maternal psychological well‐being during pregnancy by addressing childhood traumatic experiences, offering psychosocial support and incorporating integrated care approaches that bridge pre‐ and postnatal support, like parenting interventions [65], could reinforce resilience [66].

## 5 Conclusions

Although prenatal mediators, particularly maternal depression and anxiety, appear relevant to the intergenerational effects of ACEs, current evidence does not clearly establish their independent effects apart from postnatal influences. Biological pathways remain theoretically compelling but under‐investigated. Overall, more studies employing rigorous methodology to disentangle prenatal and postnatal effects are needed including integrated statistical models and intervention studies that begin in pregnancy and continue across childhood. Clarifying these temporal dynamics will be key to refining prevention strategies and informing policy recommendations.

## Author Contributions

**Sara Avendano:** conceptualization, data curation, formal analysis, methodology, writing – original draft, writing – review and editing. **Aicha Mourchidi:** data curation, formal analysis, writing – review and editing. **Susana Barbosa:** writing – review and editing. **Laetitia Davidovic:** funding acquisition, resources, writing – review and editing. **Barbara Heude:** funding acquisition, resources, writing – review and editing. **Cédric Galéra:** funding acquisition, resources, writing – review and editing. **Judith van der Waerden:** conceptualization, funding acquisition, methodology, resources, supervision, writing – original draft, writing – review and editing. All authors have read and agreed to the published version of the manuscript.

## Funding

This work was part of the EbiPsy project, financed by the French National Research Agency (Grant no. ANR‐23‐CE36‐0003‐0).

## Conflicts of Interest

The authors declare no conflicts of interest.

## Supporting information


**Table S1:** Reasons for exclusion of full‐text revised articles.

## Data Availability

No original datasets were generated for this systematic review. The full search strategy necessary to reproduce the analyses and the reasons for exclusion of studies that did not meet inclusion criteria are provided in Supporting Information. No analytic code beyond standard reference management and systematic review software was used.
