# Supplementary material for: Maternal Adverse Childhood Experiences and Child Socio‐Emotional and Behavioural Outcomes: A Systematic Review of Prenatal Mechanisms
Source: Paediatr Perinat Epidemiol. 2026 Aug 29;40(8):941–60. doi: 10.1111/ppe.70190 (PMC13763156; doi:10.1111/ppe.70190)
Supplement: Supplementary file 1 [file PPE-40-941-s001.docx]

**Supplementary material**

**Search string:**

Pubmed:

("maternal child*" OR "maternal history" OR "mother* history" OR intergenerational relation[MeSH Terms] OR intergenerational* OR inter-generational*) AND ((advers* OR trauma* OR traged* OR abus* OR household dysfunction OR illtreat* OR ill-treat* OR mal-treat* OR maltreat* OR mistreat* OR abandon* OR "ACE" OR "ACEs" OR assault* OR violen* OR neglect* OR victimi* OR aggression OR conflict OR offense* OR antipathy OR "emotional abuse" OR "psychological abuse" OR "verbal abuse" OR "physical abuse" OR "sexual abuse" OR "emotional neglect" OR "victimization" OR antipathy OR "hardship" OR depriva* OR cumulat* trauma OR polyvictimiz* ) OR ("Adverse Childhood Experiences"[Mesh] OR "Child Abuse"[Mesh] OR abandoned child[MeSH Terms] OR child abuse, sexual[MeSH Terms] OR child abus* OR child neglect* OR childhood advers* OR "childhood trauma")) AND (child* OR baby OR infant OR adolescent OR teen* OR offspring OR neonate OR newborn) AND (behavio* OR internali* OR externali* OR emotion OR peer relationship OR child temperament OR reactivity OR social withdrawal OR hyperactivity OR attention OR conduct OR maladjustment OR psychos* OR disruptive OR PTSD OR depress* OR anxi* OR mental health OR stress* OR antiso* OR psychopatology) AND (mediat* OR path* OR causal*)

WoS:

("maternal child*" OR "maternal history" OR "mother* history" OR "intergenerational relation" OR intergenerational* OR inter-generational* ) AND ((advers* OR trauma* OR traged* OR abus* OR "household dysfunction" OR illtreat* OR ill-treat* OR mal-treat* OR maltreat* OR mistreat* OR abandon* OR ACE OR ACEs OR assault* OR violen* OR neglect* OR victimi* OR aggression OR conflict OR offense* OR antipathy OR "emotional abuse" OR "psychological abuse" OR "verbal abuse" OR "physical abuse" OR "sexual abuse" OR "emotional neglect" OR victimization OR antipathy OR hardship OR depriva* OR "cumulat* trauma" OR polyvictimiz* ) OR ("Adverse Childhood Experiences" OR "Child Abuse" OR "abandoned child" OR "child abuse, sexual" OR "child abus*" OR "child neglect*" OR "childhood advers*" OR "childhood trauma" )) AND (child* OR baby OR infant OR adolescent OR teen* OR offspring OR neonate OR newborn ) AND (behavio* OR internali* OR externali* OR emotion OR "peer relationship" OR "child temperament" OR reactivity OR "social withdrawal" OR hyperactivity OR attention OR conduct OR maladjustment OR psychos* OR disruptive OR PTSD OR depress* OR anxi* OR "mental health" OR stress* OR antiso* OR psychopatology ) AND (mediat* OR path* OR causal* )

Psycinfo:

 ("maternal child*" OR "maternal history" OR "mother* history" OR "intergenerational relation" OR intergenerational* OR inter-generational* ) AND ((advers* OR trauma* OR traged* OR abus* OR "household dysfunction" OR illtreat* OR ill-treat* OR mal-treat* OR maltreat* OR mistreat* OR abandon* OR ACE OR ACEs OR assault* OR violen* OR neglect* OR victimi* OR aggression OR conflict OR offense* OR antipathy OR "emotional abuse" OR "psychological abuse" OR "verbal abuse" OR "physical abuse" OR "sexual abuse" OR "emotional neglect" OR victimization OR antipathy OR hardship OR depriva* OR "cumulat* trauma" OR polyvictimiz* ) OR ("Adverse Childhood Experiences" OR "Child Abuse" OR "abandoned child" OR "child abuse, sexual" OR "child abus*" OR "child neglect*" OR "childhood advers*" OR "childhood trauma" )) AND (child* OR baby OR infant OR adolescent OR teen* OR offspring OR neonate OR newborn ) AND (behavio* OR internali* OR externali* OR emotion OR "peer relationship" OR "child temperament" OR reactivity OR "social withdrawal" OR hyperactivity OR attention OR conduct OR maladjustment OR psychos* OR disruptive OR PTSD OR depress* OR anxi* OR "mental health" OR stress* OR antiso* OR psychopatology ) AND (mediat* OR path* OR causal* )

| **Supplementary table 1. Reasons for exclusion of full-text revised articles** | |
| --- | --- |
| **Title, author** | **Reasons for exclusion** |
| Intergenerational Pathways Linking Mothers' Adverse Childhood Experiences and Children's Social-Emotional Problems, Zhang et al. — 2021, USA | Postnatal mediation |
| Adverse Childhood Experiences in Mothers with Chronic Pain and Intergenerational Impact on Children – Dennis et al. — 2019, USA | Postnatal mediation |
| Mothers' Adverse Childhood Experiences and Their Young Children's Development. Sun et al. — 2017, USA | Postnatal mediation |
| Child maltreatment: An intergenerational cascades model of risk processes potentiating child psychopathology. Russotti et al. — 2020, USA | Postnatal mediation |
| Maternal childhood adversity and child temperament: an association moderated by child 5-HTTLPR genotype. Bouvette-Turcot et al. — 2013, Canada | No mediation |
| Maternal History of Child Maltreatment and Household Chaos: Examining the Mediating Role of Maternal and Child Psychopathology – Andrews et al. — 2022, Canada | Postnatal mediation, wrong outcome, Wrong study duration |
| Maternal Childhood Maltreatment History and Child Behavior Problems: Developmental Patterns and Mediation via Maternal Depressive Symptoms and Parenting Behavior, Harris et al. Harris et al. — 2022, USA | Postnatal mediation, Wrong study duration |
| Impact of maternal childhood trauma on child behavioral problems: The role of child frontal alpha asymmetry. van de Ven et al. — 2019, Netherlands | Postnatal mediation |
| Maternal childhood adversity and adolescent marijuana use at age 17 years: The role of parental mental health and parenting behaviors. Okine et al. — 2023, USA | Postnatal mediation, wrong outcome |
| Childhood Emotional and Conduct Problems in Childhood and Adolescence Differentially Associated with Intergenerational Maltreatment Continuity and Parental Internalizing Symptoms – Sierau et al. — 2020, USA/Germany | Postnatal mediation, wrong population |
| Early life maltreatment and depression: Mediating effect of maternal hair cortisol concentration on child abuse potential. Kluczniok et al. – 2020, Germany | Postnatal mediation, wrong outcome |
| A path model examination: maternal anxiety and parenting mediate the association between maternal adverse childhood experiences and children's internalizing behaviors. Shih et al. - 2021, USA | Postnatal mediation |
| Impact of maternal adverse childhood experiences on child socioemotional function in rural Kenya: Mediating role of maternal mental health. Rieder et al. — 2019, Kenya | Postnatal mediation |
| Maternal Experience of Abuse in Childhood and Depressive Symptoms in Adolescent and Adult Offspring: A 21-Year Longitudinal Study. Roberts et al. — 2015, USA | Postnatal mediation |
| Maternal Trauma Exposure and Childhood Anxiety Outcomes: Examining Psychosocial Mechanisms of Risk – Robinson et al. — 2016, USA | Postnatal mediation |
| Intergenerational Transmission of Trauma: Maternal Trauma-Related Cognitions and Toddler Symptoms. Fenerci et al. - 2018, USA | Postnatal mediation |
| Parents' Maltreatment Histories, Dimensions of Emotion Regulation, and Connections to Offspring Self-Regulation: A Sex-Specific Transmission Pathway. Osborne et al. — 2021, USA | Postnatal mediation |
| Maternal Childhood Sexual Trauma and Child Conduct Problems. Zvara et al. — 2017, USA | Postnatal mediation |
| Maternal adverse childhood experiences and infant subcortical brain volume. Demers et al. — 2022, USA | Postnatal mediation |
| Maternal childhood abuse and children's emotional-behavioral difficulties: Intergenerational transmission via birth outcomes and psychosocial health. Giallo et al. – 2020, Australia | Postnatal mediation |
| Maternal exposure to childhood maltreatment and mental and behavioral disorders in children. Airikka et al. - 2022, Finland | Postnatal mediation |
| Maternal Childhood Maltreatment History and Child Mental Health: Mechanisms in Intergenerational Effects – Bosquet Enlow et al. - 2018, USA | Postnatal mediation |
| Maternal Interpersonal Trauma and Child Social-Emotional Development: An Intergenerational Effect – Folger et al. — 2017, USA | Postnatal mediation, wrong exposure |
| Association between maternal adverse childhood experiences and mental health problems in offspring: An intergenerational study. Doi et al. — 2021, Japan | Postnatal mediation |
| Maternal adverse childhood experiences, child mental health, and the mediating effect of maternal depression: A cross-sectional, population-based study in rural, southwestern Uganda. Kim et al. — 2023, Uganda | Postnatal mediation |
| Maternal adverse childhood experiences, attachment style, and mental health: Pathways of transmission to child behavior problems. Cooke et al. — 2019, Canada | Postnatal mediation |
| Mediating links between maternal childhood trauma and preadolescent behavioral adjustment. Min et al. — 2012, USA | Postnatal mediation |
| Association between maternal adverse childhood experiences and child resilience and self-esteem: Results from the {K}-CHILD study. Doi et al. — 2022, Japan | Postnatal mediation |
| Developmental pathways from maternal history of childhood maltreatment and maternal depression to toddler attachment and early childhood behavioral outcomes. Alto et al.— 2018, USA | Postnatal mediation |
| Adverse Effects of Childhood Emotional Maltreatment on Child Behavioral Outcomes – Meller et al. — 2016, USA | Postnatal mediation |
| Using an adoption-biological family design to examine associations between maternal trauma, maternal depressive symptoms, and child internalizing and externalizing behaviors. Grabow et al. — 2017, USA | Postnatal mediation |
| Intergenerational continuity in child maltreatment: mediating mechanisms and implications for prevention. Berlin et al. — 2011, USA | Wrong outcome |
| Maternal early-life trauma and affective parenting style: the mediating role of HPA-axis function. Juul et al. — 2015, Denmark | Wrong outcome |
| The impact of maternal adverse childhood experiences and prenatal depressive symptoms on foetal attachment: Preliminary evidence from expectant mothers across eight middle-income countries. Brown et al.- 2021, multicountry | Wrong outcome |
| Psychological legacies of intergenerational trauma under South African apartheid: Prenatal stress predicts greater vulnerability to the psychological impacts of future stress exposure during late adolescence and early adulthood in Soweto, South Africa – Kim et al. — 2021, South Africa | Wrong outcome, wrong exposure |
| Intergenerational effects of violence on women's perinatal wellbeing and infant health outcomes: evidence from a birth cohort study in Central Vietnam. Do et al. — 2021, Vietnam | Wrong outcome |
| Hair cortisol in the perinatal period mediates associations between maternal adversity and disrupted maternal interaction in early infancy. Nystrom-Hansen et al. — 2019, Denmark/USA/UK | Wrong outcome |
| Intergenerational transmission of war-related trauma assessed 40 years after exposure. Castro-Vale et al. — 2019, Portuga | Wrong outcome, wrong exposure, wrong population |
| The impact of childhood trauma and psychophysiological reactivity on at-risk women's adjustment to parenthood. Oosterman et al. — 2018, Netherlands | Wrong outcome |
| Maternal adverse childhood experiences (ACEs) and DNA methylation of newborns in cord blood. Collender et al. — 2022, USA | Wrong outcome, wrong exposure, wrong study design |
| Stage 2 Registered Report: Epigenetic Intergenerational Transmission: Mothers' Adverse Childhood Experiences and DNA Methylation – Scorza et al. — 2023, USA | Wrong outcome |
| Maternal Exposure to Childhood Trauma Is Associated During Pregnancy With Placental-Fetal Stress Physiology – Moog et al. — 2016, USA | Wrong outcome |
| Maternal abuse history and reduced fetal heart rate variability: Abuse-related sleep disturbance is a mediator. Gustafsson et al. — 2015, USA | Wrong outcome |
| Mothers' childhood hardship forecasts adverse pregnancy outcomes: Role of inflammatory, lifestyle, and psychosocial pathways. Miller et al. – 2017, USA | Wrong outcome |
| Using network analysis to illuminate the intergenerational transmission of adversity. Hemady et al. — 2022, UK | Wrong outcome |
| The effects of childhood maltreatment on epigenetic regulation of stress-response associated genes: an intergenerational approach. Ramo-Fernández et al. — 2018, Germany | Wrong outcome |
| Maternal childhood maltreatment and perinatal outcomes: A systematic review. Souch et al. — 2022, UK | Wrong publication type |
| When one childhood meets another - maternal childhood trauma and offspring child psychopathology: A systematic review. Plant et al. — 2018, UK | Wrong publication type |
| Research Review: Intergenerational transmission of disadvantage: epigenetics and parents' childhoods as the first exposure. Scorza et al. — 2018, USA | Wrong publication type |
| Maternal History of Child Maltreatment and Household Chaos: Examining the Mediating Role of Maternal and Child Psychopathology – Andrews et al. – 2022, Canada | Wrong study design |
| Mediators and moderators in the relationship between maternal childhood adversity and children's emotional and behavioural development: a systematic review and meta-analysis. Ma et al. — 2022, multiple countries | Wrong publication type |
| Timeline of Intergenerational Child Maltreatment: the Mind-Brain-Body Interplay. Mitkovic et al. — 2017, Serbia | Wrong publication type |
| Oxytocin pathways in the intergenerational transmission of maternal early life stress. Toepfer et al. — 2016, USA/Germany | Wrong study design |
| Maternal Exposure to Adversity: Impact on the Microbiota-Gut-Brain Axis, Inflammation and Offspring Psychiatric Outcomes. Rajasekera et al. — 2021, USA | Wrong study design |
| Impact of adverse childhood experience on physical and mental health: A life-course epidemiology perspective. Fujiwara et al. — 2022, Japan | Wrong study design |
| Co-occurrence of preconception maternal childhood adversity and opioid use during pregnancy: Implications for offspring brain development. Allen et al. – 2021, USA | Wrong study design |
| A prenatal programming perspective on the intergenerational transmission of maternal adverse childhood experiences to offspring health problems. Roubinov et al. – 2021, USA | Wrong study design |
| Exploring associations between maternal adverse childhood experiences and child behavior. Stepleton et al. – 2018, USA | No mediation |
| Cultural trauma and epigenetic inheritance. Lehrner et al. – 2018, USA | Wrong publication type |
| A Developmental Psychopathology Perspective on Adverse Childhood Experiences (ACEs): Introduction to the Special Issue. Hawes et al. — 2023, USA | Wrong publication type |
| Integrating and synthesizing adversity and resilience knowledge and action: The ICARE model. Hays-Grudo et al. — 2021, USA | Wrong publication type |
| Maternal mental health and infant emotional reactivity: a 20-year two-cohort study of preconception and perinatal exposures. Spry et al. — 2018, Australia | Wrong exposure |
| Intergenerational transmission of stress: Multi-domain stressors from maternal childhood and pregnancy predict children's mental health in a racially and socioeconomically diverse, multi-site cohort. Bush et al. — 2023, USA | No mediation |
| Maternal childhood emotional abuse predicts Chinese infant behavior problems: Examining mediating and moderating processes. Liu et al. — 2019, China | Postnatal mediation |
| The perinatal origins of childhood anxiety disorders and the role of early-life maternal predictors. Galbally et al. — 2022, Australia | No mediation |
| Social buffering of the maternal and infant HPA axes: Mediation and moderation in the intergenerational transmission of adverse childhood experiences. Thomas et al. — 2021, Canada | Wrong outcome |
| A Role of Oxytocin Receptor Gene Brain Tissue Expression Quantitative Trait Locus rs237895 in the Intergenerational Transmission of the Effects of Maternal Childhood Maltreatment. Toepfer et al. — 2019, Germany/USA | Wrong outcome, no mediation |
| A Multi-Cohort Examination of the Independent Contributions of Maternal Childhood Adversity and Pregnancy Stressors to the Prediction of Children's Anxiety and Depression. Noroña-Zhou et al. — 2022, USA | No mediation |
| Intergenerational transmission of maternal childhood adversity and depression on children's internalizing problems. Roubinov et al. — 2022, USA | Postnatal mediation |
| Maternal Adverse Childhood Experience and Infant Health: Biomedical and Psychosocial Risks as Intermediary Mechanisms. Madigan et al. — 2017, USA | Postnatal mediation |
| Maternal Early Life Adversity and Infant Stress Regulation: Intergenerational Associations and Mediation by Maternal Prenatal Mental Health. Barclay et al. — 2022, USA | Wrong outcome |
| Maternal childhood abuse and neglect predicts offspring development in early childhood: The roles of reflective functioning and child sex. Garon-Bissonnette et al. — 2022, Canada | Wrong outcome |
| Associations among maternal lifetime trauma, psychological symptoms in pregnancy, and infant stress reactivity and regulation. Foss et al. — 2020, USA | Wrong exposure |
| The impacts of maternal childhood adversity, stress, and mental health on child development at 6 months in Taiwan: A follow-up study. Chang et al. — 2021, Taiwan | Wrong outcome |
| Self and Body Representation Among Women Survivors of Childhood Abuse and Its Relation to Their Infant's Temperament. Ginzburg et al. — 2026, Israel | Postnatal mediation |
| Maternal history of maltreatment interacts with DNA methylation patterns to predict infant temperament. Parks et al. — 2025, Singapore | Postnatal mediation |
| Intergenerational association of adverse childhood experiences and perinatal mental health in adult female offspring: evidence from the longitudinal grandmothers, mothers, and their Children's health in China. Wang et al. — 2025, China | Wrong outcome |
